# Kidney transplantation and perioperative complications: a prospective cohort study

**DOI:** 10.1016/j.bjane.2024.844556

**Published:** 2024-09-05

**Authors:** Priscila Sartoretto Dal Magro, Gisele Meinerz, Valter Duro Garcia, Florentino Fernandes Mendes, Maria Eugenia Cavalheiro Marques, Elizete Keitel

**Affiliations:** a Universidade Federal de Ciências da Saúde de Porto Alegre, Programa de Pós-Graduação em Patologia, Porto Alegre, RS, Brazil; b Santa Casa de Porto Alegre, Serviço de Transplante Renal, Porto Alegre, RS, Brazil; c Universidade Federal de Ciências da Saúde de Porto Alegre, Santa Casa de Porto Alegre, Programa de Residência em Anestesiologia, Porto Alegre, RS, Brazil

**Keywords:** Anesthesia, Comorbidity, Perioperative period, Kidney transplantation

## Abstract

**Background:**

Kidney transplant recipients face complex perioperative challenges due to comorbidities from chronic kidney disease. This study aimed to assess perioperative complications in kidney transplant recipients and evaluate the association between the Charlson Comorbidity Index (CCI) and complication severity using the Clavien-Dindo (CD) classification.

**Methods:**

A prospective cohort study conducted at a tertiary hospital in South Brazil from September 2020 to March 2022, including 230 adult kidney transplant recipients. Data on demographics, comorbidities, and complications were collected. Complications were categorized using the CD scale, and their relationship with CCI was analyzed using univariate and multivariate Cox regression.

**Results:**

Mean age was 49.2 ± 12.7 years, with 58.7% male recipients. The mean CCI score was 3.65 ± 1.5 points. Intraoperative complications occurred in 10.9% of patients, with notable issues including bleeding and airway difficulties. In the immediate postoperative period, 9.1% required urgent dialysis. In the 30-day follow-up, 57.8% had delayed graft function, 21.7% infections, 11.3% had vascular complications, and the mortality was 1.7%. CCI was not a significant predictor of severe complications; however, congestive heart failure was strongly associated with severe complications (HR = 6.6 95% CI 2.6–6.7, *p* < 0.001).

**Conclusions:**

Despite a low overall comorbidity profile, kidney transplant recipients faced significant perioperative challenges. The lack of a significant association between the CCI score and severe complications suggests that traditional risk assessment tools may not fully capture the risks specific to the early postoperative period in kidney transplantation, and future research should focus on developing more refined risk assessment models for chronic kidney disease patients.

## Introduction

Kidney transplantation is the most frequently performed solid organ transplant worldwide.^1^ It is the most cost-effective therapy for patients suffering from end-stage renal disease,^2^ with improved quality of life and long-term survival.^3^

Patients who undergo kidney transplant often have multiple comorbidities related either to their primary disease or to long-term chronic kidney disease,^4^ such as diabetes mellitus, peripheral vascular disease, cardiovascular disease, anemia, obesity, hyperparathyroidism, and vascular calcifications. The combination of chronic illnesses results in physiological modifications that make the perioperative phase complex, and complications are expected.^5^ The most frequent complications related to the anesthetic and surgical procedures are hemodynamic instability, airway management difficulty, bleeding and hematomas, reperfusion syndrome, thrombosis, urinary fistulas, lymphocele. In the early post-transplant period, the more common clinical complications are delayed graft function, hyperkalemia, anemia, surgical site infection, venous access infection, urinary infection, cytomegalovirus reactivation, hyperglycemia, adverse drug reactions, cardiac events and acute pulmonary edema.^6^

Reports on kidney transplant complications are infrequent and commonly presented without uniform definitions of type and severity.^7^^,^^8^ The Charlson Comorbidity Index (CCI) is a tool that assesses mortality risk over 10 years and has been used in the evaluation of kidney transplant recipients to predict risk in the early postoperative period.^9^^,^^10^ CCI is associated with severe postoperative complications, and the Clavien-Dindo (CD) scale grades their severity according to the intervention complexity needed for their management.^5^^,^^11^

The lack of standardized data on perioperative kidney transplant complications, especially national data, warrants the need for a dedicated look on patient characteristics and comorbidities. Patients with chronic kidney disease are such a particular population, and efforts to try to objectively estimate the risk of morbidity and mortality during hospitalization are important, especially for preoperative counseling of kidney transplant candidates.^10^

The primary objective of this study is to evaluate the occurrence of perioperative complications in kidney transplant recipients at our Institution. Furthermore, to evaluate the association of comorbidities, defined by CCI, and 30-day complications, as outlined by the CD classification. We hypothesize that the CCI would be significantly associated with the rate of severe perioperative complications.

## Methods

### Design

This was a prospective cohort study with consecutive adult kidney transplant recipients in a tertiary philanthropic private hospital in the South of Brazil between September 2020 and March 2022.

### Sample

The sample size was calculated according to the anticipated number of transplants in one year. Inclusion criteria were adults (> 18 years old) recipients of a kidney transplant, who agreed to participate by signing an informed consent form. Exclusion criteria was combined kidney transplant with any other organ. All sequential patients that were admitted to receive a kidney transplant during the study period were invited. Pediatric recipients and those receiving concomitant other SOT were excluded due to their specific characteristics and different risks for post-operative complications.^5^^,^^7^ There were 230 kidney transplant recipients included in the final analysis.

### Ethics

The study was approved by the local ethics committee (protocol number: 4.639.936). Patients who agreed to participate signed an informed consent form.

### Baseline data collection

Demographic data as age at transplantation, sex, Body Mass Index (BMI), cause of chronic kidney disease, type of donor, immunosuppressive therapy, Kidney Donor Profile Index (KDPI)^12^ were collected. A complete list of documented comorbidities was compiled by carefully reviewing patients’ medical records. The primary researcher personally reviewed all information and completed the CCI^13^ after the inclusion of the patient in the study.

### Procedures

All patients received standardized general anesthesia, including sevoflurane, propofol, midazolam, remifentanil or fentanyl, cisatracurium or atracurium, and 1% lidocaine. The anesthesiology team is specialized in managing the aforementioned patients and used the same anesthetic protocol for all cases. The management of the main anesthetic complications (such as airway difficulties, anaphylactic shock) was carried out following validated protocols from the American Society of Anesthesiologists (ASA)^14^ and the Brazilian Society of Anesthesiology (SBA).^15^ After kidney transplantation, all patients were transferred to the intensive care unit for anesthetic recovery and monitoring.

### Intraoperative and follow-up data

Anesthetic, surgical, and clinical complications were collected from kidney transplant day to 30 days post-transplantation, and the primary researcher actively and systematically checked the patients’ records. Collected variables included airway difficulty, bronchospasm, excessive bleeding, thrombosis, hemodynamic instability, acute myocardial infarction, acute pulmonary edema, infection, rejection. Delayed graft function was defined as the need for dialysis within the first week after transplantation, in accordance to previous publications.^16^^,^^17^ The need of dialysis for life-threatening hyperkalemia or volume overload was considered a severe complication. All patients are followed-up at our center, medical records are electronic, and inpatient and outpatient charts are integrated, as well as laboratory and imaging reports. A form was fulfilled for each patient. Afterwards, the database was double-checked by two other researchers according to the primary source.

### Severity grading

Complications were evaluated using the CD complication scale^5^ on I–V according to the level of intervention required to resolve them. A significant complication was defined as Grade II or higher, whereas Grade IIIb or higher complications were considered severe.

### Statistical analyses

All analyses were performed using SPSS® version 22.0. Continuous variables with normal distribution are presented as mean and standard deviation and compared with parametric tests. Variables with non-normal distribution are presented as median and 25‒75% interquartile interval and compared with non-parametrical tests. Categorical variables are presented as absolute and relative frequencies and compared with chi-square. Univariate and multivariate stepwise Cox regression analyses were employed to identify the risk factors linked to the development of severe postoperative complications throughout the 30 days following transplantation. Missing data were removed from the analysis. The significance level was set at *p* = 0.05.

## Results

There were 230 kidney transplant recipients included in the analysis. The baseline characteristics are presented in Table 1. The mean age was 49.2 ± 12.7 years, 135 (58.7%) were male, and the majority received organs from deceased donors (96.5%). The leading cause of renal disease was glomerulonephritis (24.3%). Most patients received induction therapy, consisting in basiliximab (49.3%) or thymoglobulin (47.4%). All patients received maintenance immunosuppression with tacrolimus, mycophenolic acid and steroids. Most patients were overweight (38.3%) or obese (20%). The median duration of hospitalization was 16.3 ± 7.8 days.

**Table 1:** Baseline characteristics of 230 kidney transplant recipients.

**Characteristics**	**n = 230 (%)**
Age, years (mean ± SD)	49.2 ± 12.7
Male gender	135 (58.7)
Cause of kidney disease	
Glomerulonephritis	56 (24.3)
Unknown	49 (21.3)
Diabetes	48 (20.9)
Hypertension	26 (11.3)
Others	26 (11.3)
Polycystic kidney disease	25 (10.9)
BMI (mean ± SD)	26.1 ± 4.6
Obese	46 (20.0)
Overweight	89 (38.7)
Deceased donor	222 (96.5)
KDPI (mean ± SD)	37.1 ± 27.4
Induction therapy	222 (96.5)
Thymoglobuline	109 (47.4)
Basiliximab	113 (49.1)
Charlson Comorbidity Index (mean ± SD) (range)	3.65 ± 1.5 (2‒9)
2	68 (29.5)
3	60 (26.0)
4	36 (15.6)
5	29 (12.6)
6	28 (12.2)
7	7 (3.0)
8	0 (‒)
9	2 (0.9)

SD, Standard Deviation; KDPI, Kidney Donor Profile Index.

### Charlson Comorbidity Index

The mean CCI was 3.65 ± 1.5 points, ranging from 2 to 9. All patients received at least two points due to chronic kidney disease, and 68 (29.5%) patients did not have any other comorbidity. Age strata accounted for 1 additional point in 77 (33.5%) and 2 points in 44 (19.1%) patients. Diabetes was the most prevalent comorbidity in 56 (24.3%) patients, followed by peripheral vascular disease in 16 (7%), myocardial infarction in 13 (5.7%) and congestive heart failure in 5 (2.2%) patients. Table S1 details CCI distribution. Table 2 displays the perioperative complications.

**Table 2:** Complications during the first 30 days after transplant surgery in 230 kidney transplant recipients.

Complications	n (%)
**Intraoperative**	25 (10.9)
1	20 (8.7)
2	5 (2.2)
Bleeding with need of hemocomponent transfusion	13 (5.6)
Airway difficulty	11 (4.7)
Bronchospasm	2 (0.8)
**Immediate Perioperative**	
Urgent dialysis (hyperkalemia or hypervolemia)	21 (9.1)
Hypotension requiring intravenous drugs	31 (13.4)
Hypertension requiring intravenous drugs	17 (7.3)
Acute pulmonary edema	5 (2.2)
**Within 30 days**	
Acute myocardial infarction	7 (3.0)
Acute cerebrovascular event	2 (0.8)
Infections	50 (21.7)
Bacterial	36 (15.6)
Viral	15 (6.5)
Fungal	1 (0.4)
Infection site (n = 50)	
Urinary	22 (9.6)
Pulmonary	15 (6.5)
Bloodstream	11 (4.8)
Abdominal	2 (0.9)
CMV	2 (0.8)
CPE	6 (2.6)
COVID-19	11 (4.7)
Acute rejection	11 (4.7)
Delay graft function	133 (57.8)
Blood components transfusion	47 (20.4)

CMV, Cytomegalovirus; CPE, Carbapenemase-Producing Enterobacteriaceae.

### Intraoperative period

Intraoperative complications occurred in 25 (10.9%) patients. Bleeding that required transfusion occurred in 13 (5.6%) patients. Airway difficulty was observed in 11 (4.7%) patients, all of them overweight/obese (*p* = 0.002). Bronchospasm occurred in 2 patients, both obese (*p* = 0.039). No cardiovascular complications occurred during the surgery.

### Immediate postoperative period

In the immediate postoperative period, 21 (9.1%) patients needed urgent dialysis for hyperkalemia or hypervolemia, considered severe complications (CD grade IVa). Vasoactive drugs were needed to treat hypotension in 31 (13.4%) patients and hypertension in 17 (7.3%). Five (2.2%) patients presented acute pulmonary edema, which was associated with pre-transplantation congestive heart failure (*p* = 0.004). Congestive heart failure was significantly associated with pre-transplantation myocardial infarction (*p* = 0.027) and cerebrovascular disease (*p* = 0.016), and was not associated with diabetes (*p* = 0.60) and obesity (*p* = 0.12).

### Thirty-day follow-up

During the 30-day postoperative period, most patients presented delayed graft function and remained dependent of dialysis (133 patients, 57.8%) in the first week. Infections occurred in 50 (21.7%) patients: 36 (15.6%) bacterial, 15 (6.5%) viral, of which 11 were COVID-19. Site of infection was urinary in 22 (9.6%), pulmonary in 15 (6.5%), bloodstream in 11 (4.8%), and abdominal in 2 (0.9%). Blood transfusion was indicated for 47 (20.4%) patients. Seven (3.0%) patients had an acute myocardial infarction, 4 with a previous history of ischemic cardiopathy (*p* < 0.001) and 3 with diabetes (*p* = 0.066). A previous ischemic cardiopathy was also associated with hypertension requiring intravenous treatment (*p* = 0.008). Eleven (4.7%) patients presented acute cellular rejection in the 30-day follow-up.

### Clavien-Dindo scores

Table 3 displays the CD grading for postoperative complication severity. All patients experienced some degree of complications, the majority Grade II (73.5%), and most presented more than one complication (n = 207; 90%). Grade II complications included need for vasoactive drugs (n = 47), hypoglycemia (n = 46), blood transfusion (n = 60), maintenance dialysis for delayed graft function (n = 133), acute rejection episodes (n = 11) and infection (n = 50). Grade III complications occurred in 27 (11.7%) patients, requiring reintervention: hematoma drainage (n = 10), graft nephrectomies due to vein thrombosis (n = 5) and arterial thrombosis (n = 3), urinary fistula (n = 4), arterial kinking (n = 2), lower urinary tract obstruction (n = 2), hematoma drainage followed by graft nephrectomy due to vein thrombosis (n = 1). Grade IV occurred in 23 (10.0%) patients, mostly urgent dialysis (n = 21), acute myocardial infarction (n = 7), acute cerebrovascular event (n = 2). Four (1.7%) patients died (Grade V) within the first 30 days post-transplantation: one from cardiovascular disease (diabetic patient), one from undetermined causes, one from major bleeding, and one from complications related to COVID-19.

**Table 3:** Frequency of patients experiencing each Clavien-Dindo grade of complication within 30 postoperative days.

Clavien-Dindo grade	Definition	n (%)
I	Any deviation from the normal postoperative course without the need for pharmacological treatment or surgical, endoscopic, and radiological interventions.	7 (3.0)
	Allowed therapeutic regimens are: drugs such as antiemetics, antipyretics, analgesics, diuretics electrolytes, and physiotherapy. This grade also includes wound infections opened at the bedside.	
II	Requiring pharmacological treatment with drugs other than such allowed for grade I complications.	169 (73.5)
	Blood transfusions and total parenteral nutrition are also included.	
III	Requiring surgical, endoscopic, or radiological intervention	27 (11.7)
IIIa	Intervention is not under general anesthesia	2 (0.9)
IIIb	Intervention under general anesthesia	25 (10.8)
IV	Life-threatening complication (including CNS complications)^a^ requiring IC/ICU management	23 (10.0)
IVa	Single organ dysfunction (including dialysis)^b^	21 (9.1)
IVb	Multiorgan dysfunction	2 (0.9)
V	Death	4 (1.7)

a Brain hemorrhage, ischemic stroke, subarachnoid bleeding, but excluding Transient Ischemic Attacks (TIA); IC, Intermediate Care; ICU, Intensive Care Unit.

b Urgent dialysis for fluid overload or severe hyperkalemia.

### Association analysis for CCI and CD

**Figure 1:**
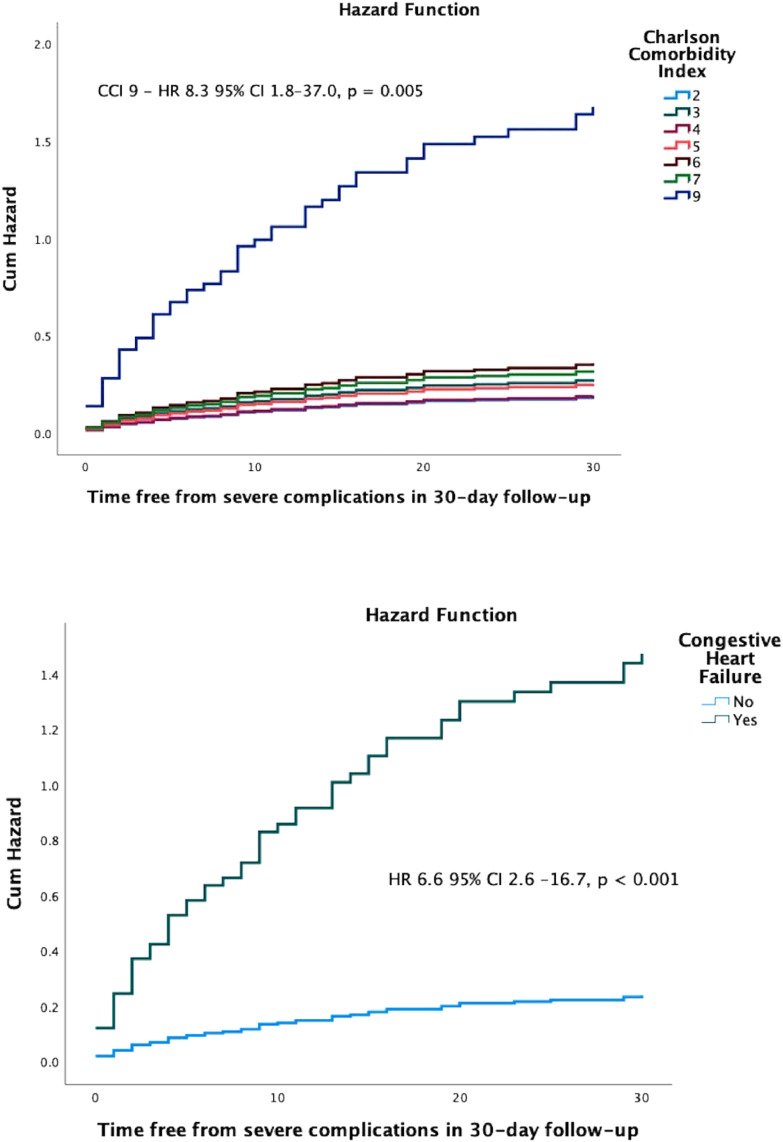
TOP: hazard function for each Charlson Comorbidity Index (CCI) and time free from severe complications (Clavien-Dindo IIIb or higher) during 30-day follow-up, adjusted for gender, body mass index, type of donor and induction therapy. BOTTOM: hazard function for congestive heart failure and time free from severe complications (Clavien-Dindo IIIb or higher) during 30-day follow-up, adjusted for age, gender, diabetes and body mass index.

Two sets of analysis of potential preexisting risk factors for severe complications (CD grade IIIb and higher, n = 52) during the first 30 days after kidney transplant were performed. The first analysis included, gender, BMI, type of donor, induction therapy and CCI, and we found significant association of CCI 9 and severe complications (Hazard Ratio [HR = 8.3], 95% CI 1.8–37.0, *p* = 0.005). In the second analysis, we tested each CCI variable, and found significant association of congestive heart failure and severe complications (HR = 7.0, 95% CI 2.0–24.0, *p* = 0.002), as shown in Figure 1. In the multivariate analysis, congestive heart failure was the only remaining variable associated with severe complications during the early postoperative period (HR = 6.6, 95% CI 2.6–16.7, *p* < 0.001), adjusted for age, gender, diabetes and BMI. This is shown in Figure 1.

The KDPI was not associated with graft loss, recipient death, or urological or vascular complications (data not shown).

## Discussion

We presented the main perioperative complications in the first 30 days after kidney transplantation in a tertiary hospital. Nearly all patients (90%) presented at least two complications, mostly graded not severe according to the Clavien-Dindo (CD) score, requiring clinical interventions. Almost 25% of patients presented severe complications, requiring surgical reinterventions or suffering life-threatening conditions and/or death. Namely, most complications were airway difficulty, bleeding with transfusion need, urgent dialysis, vasoactive drugs need, delayed graft function and infections. The surgical reintervention rate was 13% and the mortality rate was 1.7% within the initial 30 days. CCI was not associated with CD severity of complications.

All patients scored two points on the CCI because of underlying chronic kidney disease. One-third of our patients were < 50 years old and had no other prior diseases; therefore, they did not receive additional points. No patients received points for active leukemia, lymphoma, metastatic neoplasia, or acquired immunodeficiency syndrome, since that would be exclusion criteria for kidney transplant. Other studies have reported low CCI scores in chronic kidney disease patients, with diabetes as the sole and most frequent comorbidity,^5^^,^^10^ although in a higher frequency than our findings (50 vs. 25%).

Twenty-five (11%) patients experienced intraoperative complications, the most frequent of which was excessive bleeding requiring hemocomponent transfusion, as reported by other authors.^5^ Patients with chronic kidney disease often have some degree of anemia, and uremia can increase bleeding time and reveal deficient platelet function.^18^^,^^19^ Associated cardiovascular disease often warrants patients to use antiplatelet agents. Additionally, chronic kidney disease patients are prone to thrombotic events owing to increased procoagulant levels, endothelial dysfunction, advanced age, and obesity.^20^ We had one death related to excessive bleeding in the early post-kidney transplant period.

The second most common complication during surgery was airway difficulty, defined as three or more attempts at orotracheal intubation. Overall, the complication occurs in 5–15% of the patients in clinical practice and can result in morbidity or even fatality.^21^^,^^22^ Diabetes is a predictor of difficulties in airway management,^23^ since 30–40% of diabetic patients may present with a stiff joint syndrome,^24^ limiting atlanto-occipital joint mobility. Another predictor of a difficult airway is obesity, a health care problem with increasing global prevalence,^25^ in all age groups and both sexes.^26^ Overweight alters patients’ anatomy and physiology, making airway management difficult and has a potential to negatively affect respiratory function after anesthetic induction^27^ and in the postoperative period. Although our study population was predominantly overweight and 25% were diabetic, only 4.7% of patients presented this complication. This is likely due to the experienced anesthesiologists on the team and the availability of special devices for difficult airways (video laryngoscope, bougie, laryngeal mask, and fiberoptic bronchoscope) in the surgical center.^14^ No cases of anesthesia-related deaths were observed in this study. Deaths wholly attributable to anesthesia constituted the lowest incidence in most epidemiological studies, both in Brazil and abroad.^28^

Diabetes is the most frequent comorbidity in chronic kidney disease, since it is one of the major causes of kidney failure, especially in developed countries.^29^ Diabetes is associated with poor wound healing, anastomotic complications, increased risk of infection, exacerbation of ischemic damage and myocardial infarction, dehydration, and electrolyte loss.^30^ In this study, diabetes was not associated with cardiovascular events in the first 30 days after kidney transplantation, nor to severe complications (CD IIIb or higher). Other authors have described an increased risk for cardiovascular events in patients with chronic kidney disease and diabetes, specially myocardial infarction.^31^ In our pre-kidney transplant evaluation, patients with diabetes are required to undergo coronary angiography and cardiology consultation, even if asymptomatic, as part of their risk assessment.

Although cardiovascular disease was prevalent in our population (20%), no major cardiovascular events occurred during anesthesia. General anesthesia is the chosen technique for kidney transplant; however, such technique can evoke cardiorespiratory complications such as myocardial infarction and changes in pulmonary mechanics, contributing to perioperative morbidity.^32^ The rate of acute myocardial infarction (3%) and cerebrovascular (0.8%) events in the 30-day follow-up was comparable to other reports.^31^^,^^33^ One patient died of cardiovascular complications, and he had diabetes.

The most common clinical complication was delayed graft function, which was similar to other Brazilian centers.^34^^,^^35^ The high national rates of delayed graft function are due to multiple factors, specially prolonged cold ischemia time, pre-procurement deceased donor management, donor age and clinical characteristics, time on dialysis.^34^^,^^35^ Although need of dialysis is a Grade IV CD score, in the chronic kidney disease subset of patients it is not considered a severe complication, since it is a maintenance of their baseline condition.^5^ We classified dialysis need as CD IV if it was indicated for hyperkalemia or volume overload, as life-threatening situations.

Infections were the second most common cause of clinical complications in the postoperative period with no unique risk factors identified. The main infection was urinary tract infection, which is similar to previously reported data.^5^^,^^36^^,^^37^ The risk and type of infections is dependent on timing after kidney transplant. In the early postoperative period, the occurrence is primarily related to the procedure itself, to invasive devices, donor-transmitted or reactivation of latent infections, mostly viral (e.g., herpes). Bacterial infections are the most common, including antimicrobial-resistant: surgical-site, catheter-related, urinary infections, pneumonia, *Clostridium difficile* colitis.^38^ Our study was conducted during the COVID-19 pandemic, we had one death related to SARS-CoV-2 infection, acquired in the early post-kidney transplant period.

The overall incidence of surgical complications after kidney transplantation varies between 5–38%,^39^^,^^40^ and our results follow this trend (13%). The most frequent complication was hematoma, which was consistent with the need for transfusions in the perioperative period. Additionally, chronic kidney disease patients are frequently anemic and exhibit platelet dysfunction.^19^ Vascular complications were arterial and venous thrombosis (3.5%), leading to graft loss, and arterial kinking, requiring reintervention. These complications can occur at any time after transplantation, but they are particularly important in the immediate postoperative period,^1^ and the incidence was similar to other reports.^39^^,^^40^ Urological complication rates (2.6%) were comparable to other studies.^31^^,^^40^ The overall rate of surgical reintervention was higher than that reported in the USA nationwide analysis (2.2%)^41^ and similar to other reports (8–15%).^42^, ^43^, ^44^

In our study, the CCI did not predict severe CD complications in the early postoperative period, in contrast with the findings of Levine et al.^5^ Most patients had a low CCI, given an average of 3 points. As stated, the study was conducted during the COVID-19 pandemic, precluding the selection of lower-risk donors and recipients. In the multivariate analysis, only congestive heart failure was associated with severe complications, adjusted for age, obesity, and diabetes. This is different from other findings^5^ that reported an association with diabetes and peripheral vascular disease.

This study has some limitations and potential biases that should be considered when interpreting the findings. Firstly, the single-center nature is considered a weakness, but our center receives referrals from other Brazilian states to be waitlisted for kidney transplantation, which may improve the representation and the external validity of our sample. Additionally, the study was conducted during the COVID-19 pandemic, which may have influenced the selection of both donors and recipients and introduced variables that could impact the incidence of complications. Despite these limitations, the study benefits from a prospective design and systematic data collection by a dedicated research team, which enhances the reliability of the results. The strengths of this study include its comprehensive follow-up of all kidney transplant patients at one of the largest transplant centers in our country, with over 40 years of clinical experience. Future research should aim to validate these findings in diverse settings and explore how different risk factors, including those exacerbated by the pandemic, affect perioperative outcomes. Expanding studies to include multicenter data could provide a more generalizable understanding of early complications after kidney transplantation and help refine risk assessment tools for better clinical decision-making.

## Conclusions

In summary, our findings highlight that, despite a low overall comorbidity profile, kidney transplant recipients faced significant perioperative challenges, including bleeding, hypotension, and delayed graft function. Clinicians should be aware of these common complications and prepare for immediate interventions during and after surgery. The lack of a significant association between the CCI score and severe complications suggests that traditional risk assessment tools may not fully capture the risks specific to the early postoperative period in kidney transplantation. Future research should focus on developing more refined risk assessment models and exploring targeted interventions to reduce common perioperative complications and improve outcomes for kidney transplant patients.

## CRediT authorship contribution statement

**Priscila Sartoretto Dal Magro:** Conceptualization, Data curation, Investigation, Methodology, Visualization, Writing – original draft. **Gisele Meinerz:** Data curation, Formal analysis, Methodology, Visualization, Writing – original draft. **Valter Duro Garcia:** Supervision, Writing – review & editing. **Florentino Fernandes Mendes:** Supervision, Writing – review & editing. **Maria Eugenia Cavalheiro Marques:** Writing – review & editing. **Elizete Keitel:** Conceptualization, Data curation, Formal analysis, Methodology, Project administration, Supervision, Validation, Visualization, Writing – original draft.

## Conflicts of interest

The authors declare no conflicts of interest.

## Appendix Supplementary materials

Image, application 1

## Footnotes

Supplementary material associated with this article can be found in the online version at doi:10.1016/j.bjane.2024.844556.
