# Supplementary material for: Kidney transplantation and perioperative complications: a prospective cohort study
Source: Braz J Anesthesiol. 2024 Sep 5;74(6):844556. doi: 10.1016/j.bjane.2024.844556 (PMC11447349; doi:10.1016/j.bjane.2024.844556)
Supplement: Supplementary file 1 [file mmc1.docx]

**Table S1** Charlson Comorbidity Index (CCI) categories and frequency of patients in each category.

| CCI categories | Points | n (%) |
| --- | --- | --- |
| Age |  |  |
| < 50 years | 0 | 101 (43.9) |
| 50–59 years | 1 | 77 (33.5) |
| 60–69 years | 2 | 44 (19.1) |
| 70–79 years | 3 | 8 (3.5) |
| ≥ 80 years | 4 | 0 (‒) |
| Myocardial infarction | 1 | 13 (5.7) |
| Congestive heart failure | 1 | 5 (2.2) |
| Peripheral vascular disease | 1 | 16 (7.0) |
| Cerebrovascular disease | 1 | 10 (4.3) |
| Dementia | 1 | 0 (‒) |
| Chronic obstructive pulmonary disease | 1 | 4 (1.7) |
| Connective tissue disease | 1 | 14 (6.1) |
| Peptic ulcer disease | 1 | 1 (0.4) |
| Diabetes mellitus |  |  |
| Uncomplicated | 1 | 1 (0.4) |
| End-organ damage | 2 | 56 (24.3) |
| Moderate to severe chronic kidney disease | 2 | 230 (100) |
| Hemiplegia | 2 | 5 (2.2) |
| Leukemia | 2 | 0 (-) |
| Malignant lymphoma | 2 | 0 (-) |
| Solid tumor | 2 | 1 (0.4) |
| If metastatic | 6 | 0 (‒) |
| Liver disease |  |  |
| Mild | 1 | 0 (‒) |
| Moderate to severe | 3 | 1 (0.4) |
| AIDS | 6 | 0 (‒) |

AIDS, Acquired Immunodeficiency Syndrome.
